# Factors associated with postoperative delirium in patients undergoing oral and maxillofacial surgery: a meta-analysis of observational study

**DOI:** 10.3389/fmed.2026.1795458

**Published:** 2026-04-28

**Authors:** Yifan Lin, Xiaofen Chen, Lulu Lin, Ruibin Zheng

**Affiliations:** School & Hospital of Stomatology, Wenzhou Medical University, Wenzhou, China

**Keywords:** general anesthesia, meta-analysis, oral and maxillofacial surgery, postoperative delirium, risk factors, systematic review

## Abstract

**Background:**

Postoperative delirium (POD) is a common and serious complication, particularly prevalent among patients undergoing oral and maxillofacial surgery (OMFS). Although studies have explored risk factors for POD, a systematic assessment for OMFS patients remains lacking. This study aimed to identify and synthesize factors associated with POD in patients undergoing OMFS. However, the currently available evidence appears to be derived mainly from major oncologic and reconstructive OMFS populations.

**Methods:**

The literature search was conducted from the establishment of the database to December 10, 2025, covering PubMed, Embase, Cochrane Library, and Web of Science. All studies involving POD in patients undergoing OMFS were included in the analysis. The quality of included studies was assessed using the Newcastle-Ottawa Scale (NOS). Data analysis was performed using Stata 15 software, employing a random-effects model for meta-analysis of potential risk factors.

**Results:**

A total of seven studies involving 2,398 patients were included. The meta-analysis showed that age >65 years [OR = 1.63, 95% CI (1.15, 2.31)], male sex [OR = 1.90, 95% CI (1.17, 3.09)], alcohol consumption [OR = 3.43, 95% CI (2.09, 5.66)], smoking [OR = 2.34, 95% CI (1.19, 4.59)], and insomnia [OR = 2.27, 95% CI (1.24, 4.19)] were associated with POD in patients undergoing OMFS.

**Conclusion:**

This study suggests that age >65 years, male sex, alcohol consumption, smoking, and insomnia may be associated with POD in patients undergoing OMFS. However, the available evidence is derived predominantly from major oral cancer and reconstructive surgery populations, and all included studies were observational. Therefore, these findings should be interpreted as associations rather than causal risk factors, and their applicability to the broader spectrum of OMFS procedures remains uncertain.

**Systematic review registration:**

https://www.crd.york.ac.uk/PROSPERO/view/CRD420251267779

## Background

Postoperative delirium (POD) refers to an acute, reversible disturbance of consciousness occurring after surgery, typically accompanied by cognitive changes such as inattention, confusion, and disorientation (1). The occurrence of delirium not only imposes a significant burden on patients' physical health but may also impair recovery, prolong hospital stays, increase medical costs, and is associated with higher in-hospital mortality rates (2). Although delirium is commonly perceived as a complication predominantly affecting the elderly, its incidence and impact across other age groups cannot be overlooked (3). Particularly among patients undergoing oral and maxillofacial surgery(OMFS), the occurrence of delirium has garnered increasing clinical attention due to the unique nature of these procedures and the challenges of postoperative recovery (4).

OMFS primarily addresses diseases of the oral cavity, maxillofacial region, and associated tissues (5). Common procedures include tumor resection, jaw reconstruction, and oral rehabilitation. While most of these surgeries are classified as non-major, postoperative complications such as pain, bleeding, infection, and delirium remain significant challenges in clinical management (6). POD often carries severe psychological and physiological consequences (7). Patients may exhibit emotional instability, behavioral disturbances, or even violent tendencies, which not only complicate nursing care but may also impair postoperative recovery and rehabilitation (8).

Despite numerous studies exploring delirium mechanisms and influencing factors, research on POD risk factors specific to OMFS patients remains scarce (9, 10). Existing studies predominantly focus on elderly populations undergoing traditional surgical procedures, with limited data available for OMFS patients (11, 12). Consequently, understanding in this field remains in its preliminary stages. Oral and maxillofacial surgeries are typically associated with significant local pain, anesthetic reactions, and prolonged postoperative recovery periods. These factors may all serve as potential triggers for POD (13). Therefore, identifying risk factors influencing POD in OMFS patients holds significant importance for clinical management and prevention (14).

Current research on POD primarily focuses on factors such as medication use, preoperative health status, and surgical type. Studies indicate that advanced age, preoperative cognitive impairment, infection, malnutrition, and medication use (sedatives, anesthetics) are established risk factors for POD (15). Additionally, postoperative pain, surgical complications, and the complexity of postoperative recovery are recognized as major contributors to delirium (16). For patients undergoing OMFS, certain unique factors—such as surgical trauma to the oral and maxillofacial regions, postoperative bleeding, and the localized effects of anesthesia on these areas—may also constitute additional risk factors (17).

Several systematic reviews and meta-analyses (18, 19) have evaluated postoperative delirium or postoperative cognitive dysfunction in broader perioperative settings, including patients undergoing major non-cardiac surgery, orthopedic surgery, and procedures performed under general or regional anesthesia. These studies have identified a range of perioperative risk factors, but their findings may not be directly generalizable to OMFS, which involves distinct surgical trauma, airway management challenges, reconstructive procedures, and postoperative recovery characteristics. To date, a focused quantitative synthesis of POD risk factors specifically in OMFS patients remains limited.

Although OMFS encompasses a broad range of procedures, including tumor surgery, trauma surgery, and oral rehabilitation, the currently available literature on postoperative delirium appears to be concentrated mainly in patients undergoing major oral cancer surgery, head and neck tumor surgery, and reconstructive procedures such as free flap reconstruction. Therefore, while this review was designed to address OMFS broadly, the available evidence may primarily reflect higher-complexity oncologic and reconstructive surgical populations rather than the full spectrum of OMFS procedures.

## Methods

This systematic review and meta-analysis were conducted in accordance with the PRISMA 2020 statement (20). And it is registered in Prospero with registration number CRD420251267779.

### Inclusion and exclusion criteria

#### Inclusion criteria

##### Study type

This study includes clinical research such as cohort studies and case-control studies.

##### Patient population

The study population comprised adult patients undergoing OMFS, regardless of specific disease type (tumors, trauma, or dental restoration). However, we anticipated that the available evidence might be unevenly distributed across OMFS subtypes, depending on the existing literature.

##### POD diagnosis

Included studies must report explicit diagnostic criteria for POD and utilize recognized diagnostic tools (e.g., DSM-IV, DSM-5, ICD-10) for assessment.

##### Data completeness

Studies must provide sufficient patient information, including preoperative baseline characteristics (age, gender), POD incidence rates, and assessment data on associated risk factors.

#### Exclusion criteria

Studies involving subjects who are not OMFS patients (only involving patient groups from other surgical fields) were excluded.

Studies lacking delirium diagnostic criteria: Studies that did not explicitly use recognized diagnostic tools or lacked delirium-related diagnostic criteria were excluded.

##### Interventional studies

Clinical trials primarily focused on interventions (drug trials, psychological intervention trials) were excluded if their content was not directly related to POD risk factors.

##### Studies of low quality

Research with serious methodological flaws (insufficient sample size, incomplete data, inappropriate analytical methods) or studies whose design precluded valid conclusions were excluded. Studies overlapping with other literature using identical samples or data were retained only if they represented the most representative or highest-quality literature. All studies based on individual case reports or case series were excluded.

### Literature retrieval

Two researchers conducted independent systematic searches of the following databases: PubMed, Web of Science, Embase, and the Cochrane Library. The search period for all databases was from the inception of each database to December 10, 2025. The search strategy combined Medical Subject Headings (MeSH) with free-text terms, consisting of three main components: “POD”, “Oral”, “Surgery” and “risk factor.” The specific search strategy is detailed in Supplementary Material Table S1. The search strategy was adjusted appropriately for each database based on its characteristics. To further ensure the comprehensiveness of the literature, this study also manually searched the reference lists of included studies to supplement any potentially overlooked relevant research. In cases of disagreement during the search and screening process, a third researcher was involved to mediate and resolve the issue.

### Study selection

During the literature screening process, two researchers independently used EndNote 21 software to initially screen the literature obtained from the search, first through the titles and abstracts, and then to exclude literature that clearly did not meet the inclusion criteria. Subsequently, the remaining literature was reviewed by reading the full text in its entirety to further determine whether it met the inclusion and exclusion criteria. Any disagreements between the two researchers were resolved through discussion and negotiation; if consensus could not be reached, a third researcher was consulted for adjudication.

### Data extractions

This study was conducted by two researchers who independently extracted relevant data from the eligible literature using an Excel sheet based on the inclusion criteria. The extraction included the basic information of the study (first author, year of publication, country and study design), the basic characteristics of the study population (sample size, number of POD, gender, and mean age, Diagnostic criteria for POD), regression analysis, In the process of data extraction, if two investigators disagreed on the extracted data, the issue was resolved through discussion, and if no agreement was reached, a third investigator adjudicated.

### Quality evaluation

The risk of bias of the included studies was independently assessed by two investigators, and the results were cross-checked. For cohort and case-control studies, the Newcastle–Ottawa Scale (NOS) was used to assess methodological quality (21). The NOS evaluates studies based on three dimensions: population selection, comparability, and exposure or outcome, with eight items totaling nine points. Scores range from 0 to 4 (low quality), 5 to 6 (moderate quality), and 7 to 9 (high quality).

### Statistical analysis

Statistical analyses were performed using Stata 15.0. Odds ratios (ORs) with 95% confidence intervals (CIs) were used as the summary effect measures. ORs were selected because the included studies were observational in design, primarily cohort and case-control studies, and the original studies either reported ORs directly or provided data suitable for OR-based pooling. A random-effects model was applied to account for potential between-study heterogeneity. Statistical heterogeneity was assessed using the I^2^ statistic and Cochran's *Q*-test. An I^2^ value >50% was considered to indicate substantial heterogeneity. Sensitivity analyses were performed when necessary to evaluate the robustness of the pooled estimates. Publication bias was assessed using funnel plots and Egger's test when an adequate number of studies were available. If indicated, the trim-and-fill method was considered for further exploration of potential publication bias.

## Results

### Literature screening results

**Figure 1:**
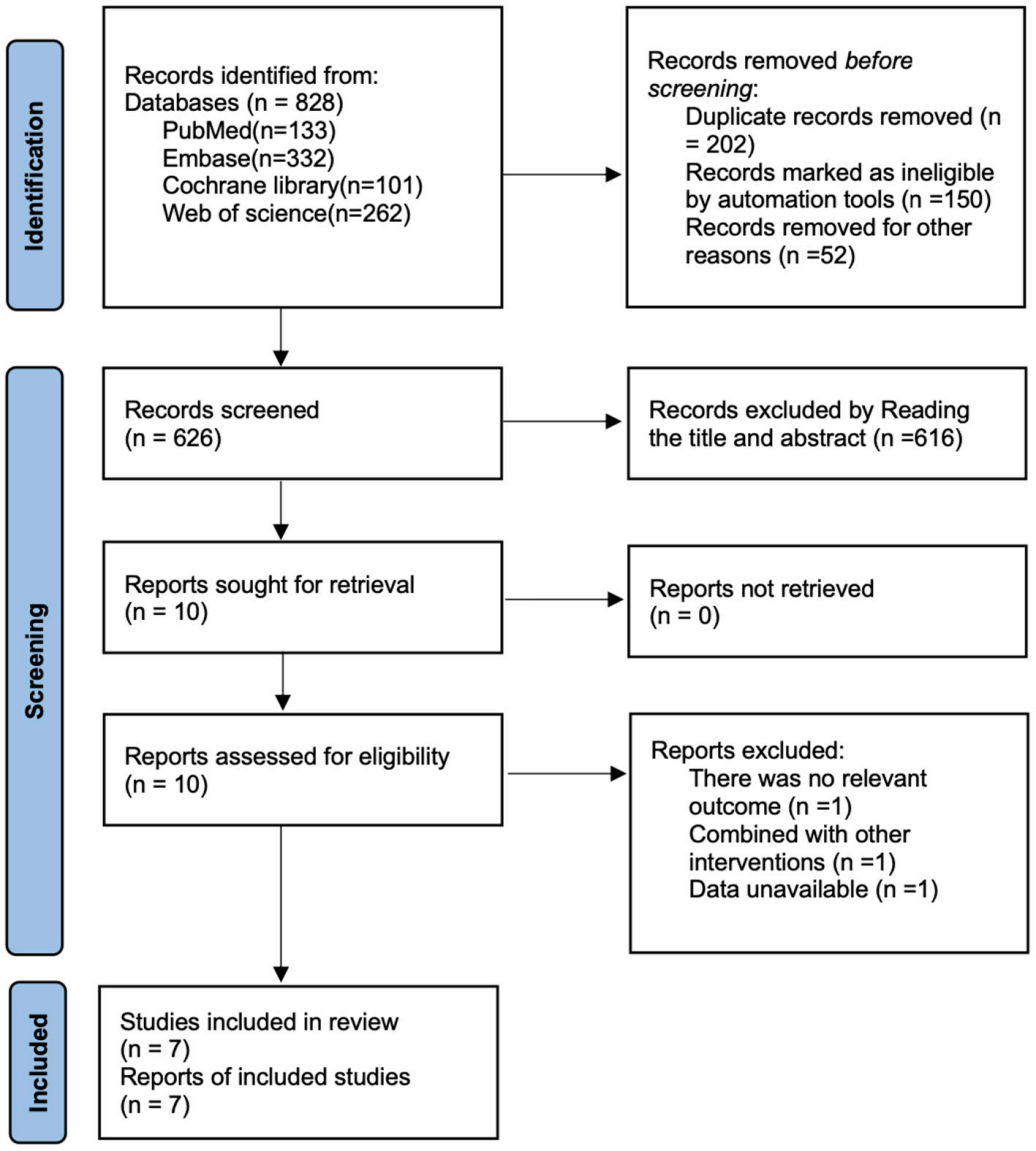
Flow chart of literature screening.

As shown in Figure 1, a total of 828 articles were retrieved from PubMed (*n* = 133), Embase (*n* = 332), Cochrane Library (*n* = 101), and Web of Science (*n* = 262), a total of 828 articles were identified. After removing 202 duplicate records, 616 articles were excluded based on title and abstract screening. Full-text review eliminated studies with no relevant outcomes (*n* = 1), combined with other interventions (*n* = 1), or unavailable data (*n* = 1). Ultimately, seven articles (22–28) were included for analysis.

### Basic characteristics of the included studies

This study included a total of seven research articles involving 2,398 patients, with a POD incidence of 15.5% (371/2,398). Among these, two studies were case-control studies and 5 were cohort studies. The age range was 51–68 years, with specific baseline characteristics presented in Table 1. Although the review eligibility criteria covered oral and maxillofacial surgery broadly, most of the included studies were conducted in patients undergoing major oral cancer surgery, head and neck oncologic surgery, or reconstructive procedures such as free flap reconstruction, with limited representation of other OMFS subtypes.

**Table 1:** Table of basic characteristics of included literature.

Author	Year	Study design	Country	Sample size	Number of deliriums	Gender (M/F)	Mean age	Diagnosis of delirium	Regression model
Chen	2025	cohort study	China	252	70	134/118	68	American Psychiatric Association's Diagnostic and Statistical Manual of Mental Disorders for criteria	Logistic regression
Crawford	2021	cohort study	Glasgow	1006	75	668/413	65.41	NR	Logistic regression
Densky	2019	cohort study	Canada	515	56	341/174	60.1	Diagnostic and Statistical Manual of Mental Disorders (Fourth Edition)	Logistic regression
Hasegawa	2015	cohort study	Japan	188	29	110/78	54.5	Diagnostic and Statistical Manual of Mental Disorders (Fourth Edition)	Logistic regression
Makiguchi	2020	cohort study	Japan	122	45	82/40	59.9	Diagnostic and Statistical Manual of Mental Disorders (Fourth Edition)	Logistic regression
Ooms	2023	case-control	Germany	110	55	69/41	68	Diagnostic and Statistical Manual of Mental Disorders (Fourth Edition)	Logistic regression
Ortner	2021	case-control	Germany	205	41	100/105	51.4	Diagnostic and Statistical Manual of Mental Disorders (Fourth Edition)	Logistic regression

### Risk of bias results

This study evaluated article quality using the NOS score. The results (Table 2) indicate that two articles scored 8 scores, and five articles scored 9 scores. In summary, all included studies were of high quality.

**Table 2:** NOS scores results.

Cohort study									
Study	Representativeness of the exposed group	Selection of non-exposed groups	Determination of exposure factors	Identification of outcome indicators not yet to be observed at study entry	Comparability of exposed and unexposed groups considered in design and statistical analysis	Design and statistical analysis	Adequacy of the study's evaluation of the outcome	Adequacy of follow-up in exposed and unexposed groups	Total scores
Chen 2025	^*^	^*^	^*^	^*^	^**^	^*^	^*^	^*^	9
Crawford 2021	^*^	^*^	^*^	/	^**^	^*^	^*^	^*^	8
Densky 2019	^*^	^*^	^*^	/	^**^	^*^	^*^	^*^	8
Hasegawa 2015	^*^	^*^	^*^	^*^	^**^	^*^	^*^	^*^	9
Makiguchi 2020	^*^	^*^	^*^	^*^	^**^	^*^	^*^	^*^	9
Case control									
Ooms 2023	^*^	^*^	^*^	^*^	^**^	^*^	^*^	^*^	9
Ortner 2021	^*^	^*^	^*^	/	^**^	^*^	^*^	^*^	8

^*^means one score.

^**^ means two scores.

### Meta-analysis results

#### Age>65years

**Figure 2:**
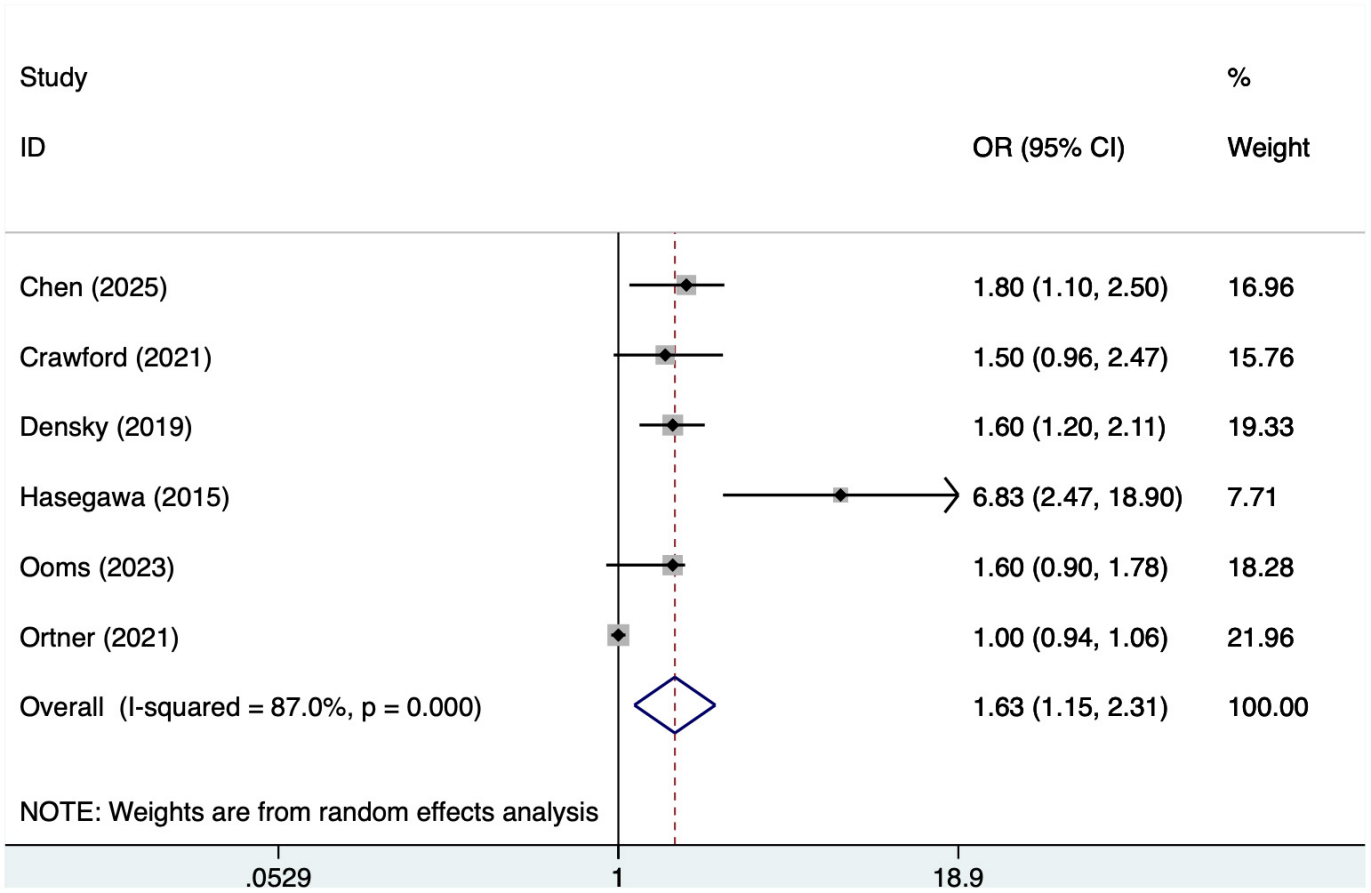
Forest plot of meta-analysis for age>65years.

Six studies reported age >65 years, with significant heterogeneity (*I*^2^ = 87%, *P* = 0.001). Analysis results (Figure 2) indicate that age >65 is associated with POD in OMFS patients [OR = 1.63, 95% CI (1.15, 2.31)]. Sensitivity analysis (Supplementary Material Figure S1) confirms the robustness of this finding, which is not influenced by any single study.

#### Male

**Figure 3:**
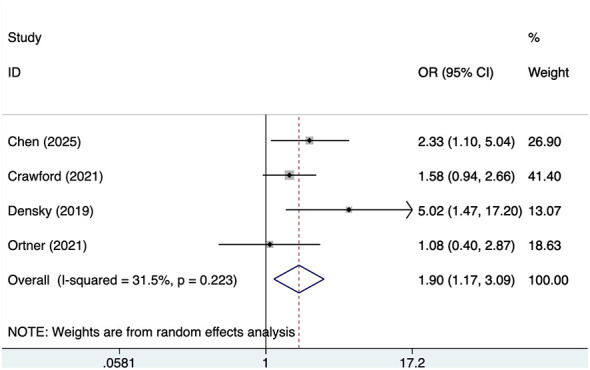
Forest plot of meta-analysis for male.

Four studies reported male, with no significant heterogeneity (*I*^2^ = 31.5%, *P* = 0.223). Analysis results (Figure 3) indicate that male is associated with POD in OMFS patients [OR = 1.90, 95% CI (1.17, 3.09)].

#### Alcohol

**Figure 4:**
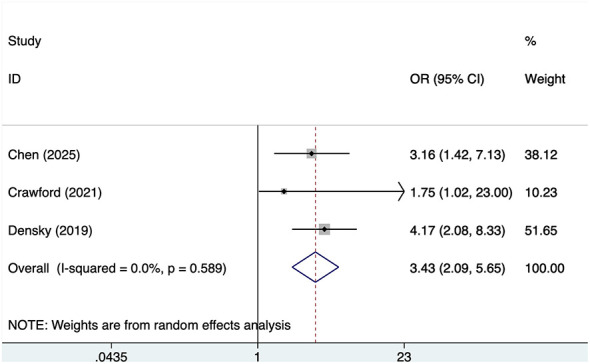
Forest plot of meta-analysis for alcohol.

Three studies reported alcohol, with no significant heterogeneity (*I*^2^ = 0%, *P* = 0.589). Analysis results (Figure 4) indicate that alcohol is associated with POD in OMFS patients [OR = 3.43, 95% CI (2.09, 5.66)].

#### Smoking

**Figure 5:**
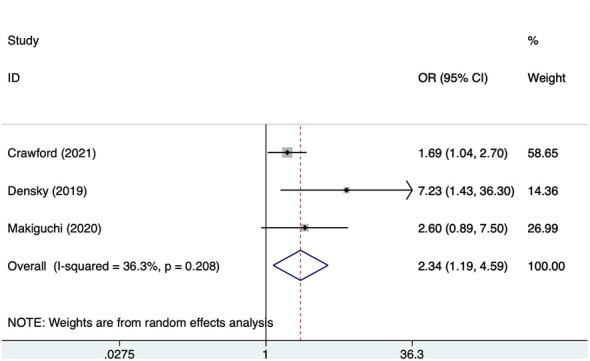
Forest plot of meta-analysis for smoking.

Three studies reported smoking, with no significant heterogeneity (*I*^2^ = 36.3%, *P* = 0.208). Analysis results (Figure 5) indicate that smoking is associated with POD in OMFS patients [OR = 2.34, 95% CI (1.19, 4.59)].

#### Insomnia

**Figure 6:**
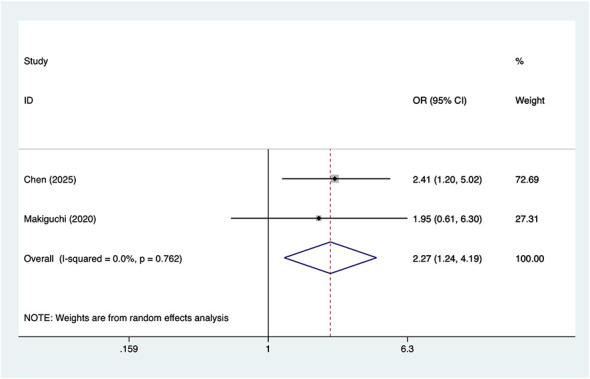
Forest plot of meta-analysis for insomnia.

Two studies reported insomnia, with no significant heterogeneity (*I*^2^ = 0%, *P* = 0.762). Analysis results (Figure 6) indicate that insomnia is associated with POD in OMFS patients [OR = 2.27, 95% CI (1.24, 4.19)].

#### Publication bias

This study employed funnel plots to detect publication bias. Results (Supplementary Materials Figures S2–S3) indicate that the funnel plot for age >65 years exhibits asymmetry, suggesting a higher likelihood of publication bias. In contrast, the funnel plot for males is symmetric, indicating a lower likelihood of publication bias. Due to the limited number of studies, no assessment of publication bias was conducted for alcohol, smoking, or insomnia.

Because of the small number of studies, it was not feasible to conduct reliable subgroup analyses or meta-regression to further investigate the sources of heterogeneity.

## Discussion

This systematic review and meta-analysis examined factors associated with postoperative delirium in patients undergoing OMFS. However, it should be noted that the currently available evidence was derived predominantly from studies of major oral cancer surgery, head and neck oncologic surgery, and reconstructive procedures, rather than the full spectrum of OMFS interventions. Therefore, the present findings may be most applicable to these higher-complexity surgical populations and should not be generalized uncritically to all OMFS patients.

This meta-analysis suggested that age >65 years was associated with postoperative delirium in patients undergoing oral and maxillofacial surgery. This finding is broadly consistent with previous literature (29). Indicating that older patients may be more vulnerable to delirium because of age-related physiological decline, reduced cognitive reserve, and greater susceptibility to perioperative stress (30). However, this result should be interpreted cautiously because substantial heterogeneity was observed across studies (*I*^2^ = 87%). Several factors may account for this variability. First, the included studies likely differed in patient characteristics, including baseline age structure, comorbidity burden, frailty status, nutritional condition, and underlying disease severity. Second, the types and complexity of surgery may have varied considerably, with some studies focusing mainly on major oral cancer or head and neck reconstructive procedures, which may carry different delirium risks compared with less extensive OMFS procedures. Third, perioperative management may have differed across studies, including anesthesia protocols, postoperative analgesia, ICU admission practices, sedation exposure, and the intensity of postoperative monitoring and support. Fourth, variation in delirium assessment approaches, including differences in diagnostic criteria, screening tools, timing of assessment, and assessor training, may also have contributed to between-study inconsistency. Taken together, these factors may explain the high heterogeneity observed for age, and the pooled estimate should therefore be interpreted as a general signal of association rather than a precise effect size applicable to all OMFS settings.

Regarding the relationship between gender and POD, the findings of this study indicate that male patients have a higher risk of POD. Although the four studies showed no significant heterogeneity (*I*^2^ = 31.5%), suggesting relative consistency in this result, the mechanism underlying gender as a potential risk factor for POD remains complex. Male patients may exhibit distinct behavioral patterns and health statuses compared to females before, during, and after surgery (31). For instance, male patients may consume higher levels of alcohol and often carry a greater burden of chronic diseases, factors that could indirectly influence their postoperative recovery (32). Gender may serve primarily as an indirect influence, primarily through associations with delirium via other health behaviors such as alcohol consumption and smoking (33). We recommend that clinicians, while acknowledging the higher risk of delirium in male patients, should comprehensively evaluate their overall health status and other contributing factors.

This meta-analysis found that alcohol consumption and smoking were each associated with higher odds of postoperative delirium in patients undergoing oral and maxillofacial surgery. Although these findings may have clinical relevance for perioperative risk assessment, they should be interpreted cautiously because each pooled analysis was based on only a small number of studies. In addition, the present review does not allow conclusions regarding whether alcohol use and smoking exert independent, overlapping, or interacting effects on POD. While these exposures may plausibly be related to perioperative vulnerability through broader effects on neurologic, cardiovascular, or general health status, such explanations were not directly examined in the included studies. Therefore, these interpretations should be considered speculative and hypothesis-generating rather than definitive.

This study found that both alcohol consumption and smoking were significantly associated with the risk of POD in patients undergoing OMFS. Specifically, a history of alcohol consumption was associated with an increased risk of POD (OR=3.43), and smoking also significantly increased the risk of POD (OR=2.34). Both factors showed high consistency across existing literature and no significant heterogeneity (alcohol: *I*^2^ = 0%; smoking: *I*^2^ = 36.3%), indicating their effects on POD are broadly consistent. Alcohol consumption and smoking are both common lifestyle-related exposures and were each associated with higher odds of POD in the present meta-analysis. However, whether these two factors exert independent, overlapping, or potentially interacting effects on POD cannot be determined from the available data and requires further investigation (34). Patients with long-term alcohol consumption and smoking histories typically exhibit chronic damage to neurological and cardiovascular systems (35, 36). Such damage not only affects anesthetic response and postoperative recovery but may also exacerbate POD by altering cognitive function and emotional states. Furthermore, alcohol consumption and smoking are closely associated with multiple chronic diseases (37). The presence of these conditions may complicate postoperative recovery, increase the risk of surgical complications, and consequently elevate the likelihood of POD.

Therefore, in clinical practice, assessing alcohol consumption and smoking habits should be integrated into the overall health evaluation of patients, particularly during the preoperative preparation phase. Clinicians should focus on evaluating patients' histories of alcohol use and smoking, and implement comprehensive interventions tailored to their individual health status and other potential risk factors. For patients with these habits, thorough health education and lifestyle interventions should be conducted preoperatively to minimize their impact on postoperative recovery. Simultaneously, postoperatively, enhanced monitoring of cognitive function and mental status is essential for these patients to promptly identify early signs of POD and initiate appropriate therapeutic interventions.

The association between insomnia and POD was also preliminarily validated in this study. However, as only two studies have examined the relationship between insomnia and POD, caution should be exercised when interpreting these findings. Insomnia may exacerbate POD through multiple mechanisms, particularly when patients already experience preoperative emotional issues such as anxiety or depression (38). In such cases, insomnia may further deteriorate the patient's mental state, leading to POD. However, current research on the relationship between insomnia and POD remains limited and subject to certain constraints. Therefore, we recommend that future studies expand sample sizes to further explore the impact of insomnia on POD.

### Strengths and limitations

This study has several strengths, including a structured systematic review process, PROSPERO registration, PRISMA-based reporting, and quantitative synthesis of the currently available evidence. However, several limitations should be considered. First, only seven studies were included, and some pooled analyses were based on only a small number of studies. Second, substantial heterogeneity was observed for certain outcomes. Third, all included studies were observational in design, so the pooled findings should be interpreted as associations rather than causal relationships. Fourth, although the eligibility criteria covered OMFS broadly, most included studies focused on major oral cancer surgery, head and neck oncologic procedures, and reconstructive surgery, which may limit the applicability of the findings to the full range of OMFS procedures. Therefore, caution is warranted when generalizing these results to lower-complexity or non-oncologic OMFS populations.

## Conclusion

This systematic review and meta-analysis suggest that age >65 years, male sex, alcohol consumption, smoking, and insomnia may be associated with postoperative delirium in patients undergoing OMFS. However, these findings should be interpreted cautiously. The current evidence base is limited, all included studies were observational, and the available data were derived predominantly from major oral cancer, head and neck oncologic, and reconstructive surgical populations rather than the full range of OMFS procedures. Accordingly, these factors should be regarded as associated variables rather than definitive causal risk factors. They may help identify patients at potentially increased risk of POD, but further large-scale prospective studies across a broader range of OMFS settings are needed before firm causal or preventive conclusions can be drawn.

## Data Availability

The original contributions presented in the study are included in the article/Supplementary material, further inquiries can be directed to the corresponding author.
