# Supplementary material for: Factors associated with postoperative delirium in patients undergoing oral and maxillofacial surgery: a meta-analysis of observational study
Source: Front Med (Lausanne). 2026 Apr 28;13:1795458. doi: 10.3389/fmed.2026.1795458 (PMC13160724; doi:10.3389/fmed.2026.1795458)
Supplement: Supplementary file 1 [file Data_Sheet_1.docx]

Table S1 PubMed specific search strategy

((("Mouth"[Mesh]) OR ((((((((((((((Mouth[Title/Abstract]) OR (Cavitas Oris[Title/Abstract])) OR (Oral Cavity[Title/Abstract])) OR (Cavity, Oral[Title/Abstract])) OR (Vestibule of the Mouth[Title/Abstract])) OR (Vestibule Oris[Title/Abstract])) OR (Oral Cavity Proper[Title/Abstract])) OR (Cavitas oris propria[Title/Abstract])) OR (Mouth Cavity Proper[Title/Abstract])) OR (Oral Cancer[Title/Abstract])) OR (Oral[Title/Abstract])) OR (Dental[Title/Abstract])) OR (free-flap reconstruction[Title/Abstract])) OR (oral tumour[Title/Abstract]))) AND (("Delirium"[Mesh]) OR (((((((((((((Delirium[Title/Abstract]) OR (Delirium of Mixed Origin[Title/Abstract])) OR (Mixed Origin Delirium[Title/Abstract])) OR (Mixed Origin Deliriums[Title/Abstract])) OR (Subacute Delirium[Title/Abstract])) OR (Deliriums, Subacute[Title/Abstract])) OR (Delirium, Subacute[Title/Abstract])) OR (Subacute Deliriums[Title/Abstract])) OR (Postoperative Delirium[Title/Abstract])) OR (Delirium, Postoperative[Title/Abstract])) OR (Post-Operative Delirium[Title/Abstract])) OR (Delirium, Post-Operative[Title/Abstract])) OR (Post Operative Delirium[Title/Abstract])))) AND (("Risk Factors"[Mesh]) OR ((((((((((((((((((((Risk Factors[Title/Abstract]) OR (Factor, Risk[Title/Abstract])) OR (Risk Factor[Title/Abstract])) OR (Population at Risk[Title/Abstract])) OR (Populations at Risk[Title/Abstract])) OR (Risk Scores[Title/Abstract])) OR (Risk Score[Title/Abstract])) OR (Score, Risk[Title/Abstract])) OR (Risk Factor Scores[Title/Abstract])) OR (Risk Factor Score[Title/Abstract])) OR (Score, Risk Factor[Title/Abstract])) OR (Health Correlates[Title/Abstract])) OR (Correlates, Health[Title/Abstract])) OR (Social Risk Factors[Title/Abstract])) OR (Factor, Social Risk[Title/Abstract])) OR (Factors, Social Risk[Title/Abstract])) OR (Risk Factor, Social[Title/Abstract])) OR (Risk Factors, Social[Title/Abstract])) OR (Social Risk Factor[Title/Abstract])) OR (Predictors[Title/Abstract])))


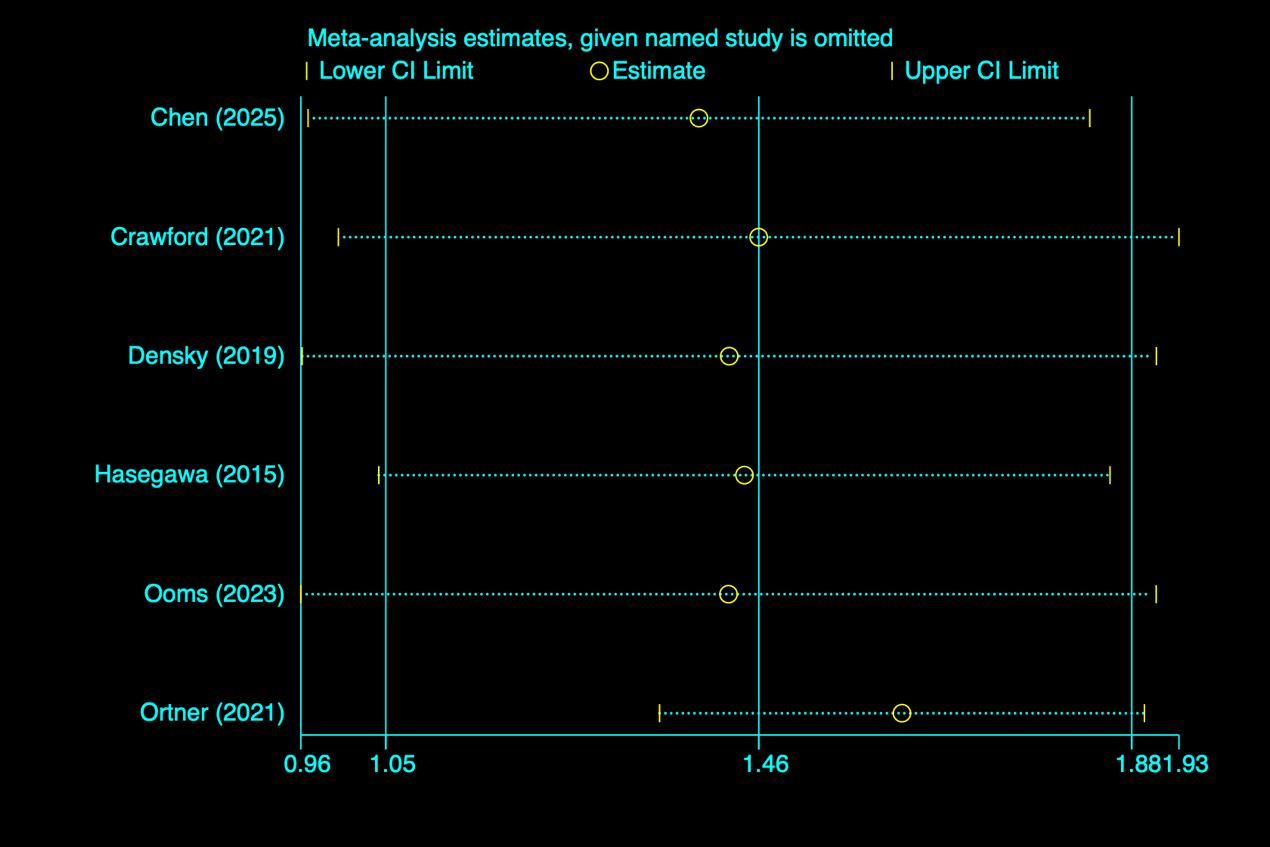


Figure S1 Sensitivity analysis of age>65


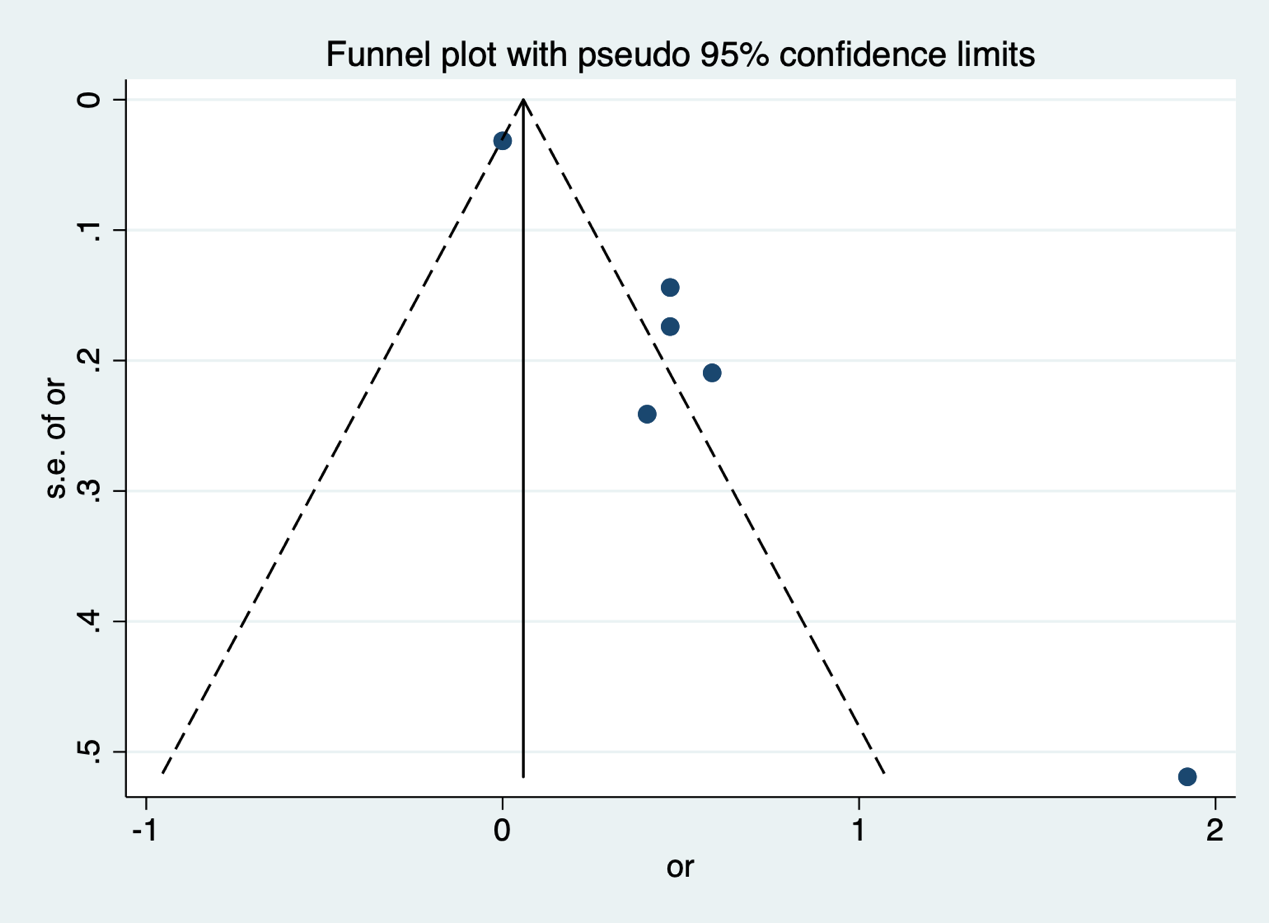


Figure S2 Funnel plot of meta-analysis of age>65


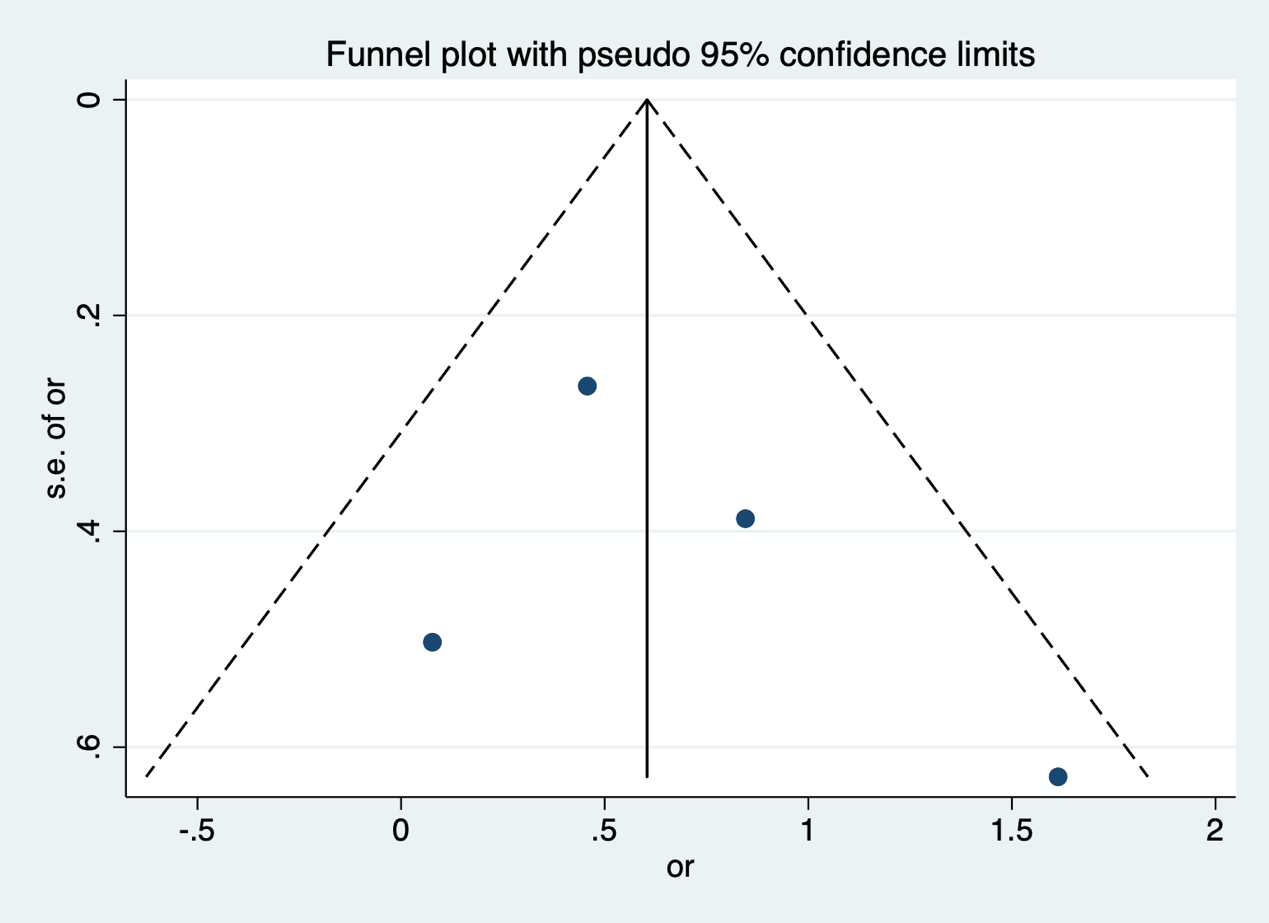


Figure S3 Funnel plot of meta-analysis of male
